# Projecting Current and Future Habitat Suitability of the Pepper Weevil, *Anthonomus eugenii* Cano, 1894 (Coleoptera: Curculionidae), in China: Implications for the Pepper Industry

**DOI:** 10.3390/insects16020227

**Published:** 2025-02-19

**Authors:** Qisong Li, Jianxiang Mao, Weifeng Wang, Ruijun Liu, Qiufan Xie, Shiyao Su, Zhong Wang, Yunzhe Song, Yongcong Hong, Pumo Cai

**Affiliations:** 1 College of Tea and Food Science, Wuyi University, Wuyishan 354300, China; liqisong0591@126.com (Q.L.); maojianxiang@wuyiu.edu.cn (J.M.); 52403030050@fafu.edu.cn (W.W.); xieqiufan@wuyiu.edu.cn (Q.X.); sushiyao@wuyiu.edu.cn (S.S.); 52303030078@fafu.edu.cn (Z.W.); songyunzhe@wuyiu.edu.cn (Y.S.); 2 College of Horticulture, Fujian Agriculture and Forestry University, Fuzhou 350001, China; 3 College of Mathematics and Computer Science, Wuyi University, Wuyishan 354300, China; mengliu@wuyiu.edu.cn; 4 Key Laboratory of Biopesticide and Chemical Biology, Ministry of Education, Fujian Agriculture and Forestry University, Fuzhou 350001, China

**Keywords:** climate change, pepper weevil, geographical distribution, maximum entropy model, centroid shift

## Abstract

The pepper weevil, *Anthonomus eugenii* Cano, 1894 (Coleoptera: Curculionidae), poses a significant threat to pepper cultivation, causing extensive crop damage and economic losses. While numerous studies have addressed its occurrence, biology, and control methods, less attention has been given to how climate change might alter its distribution. This research utilized the optimized MaxEnt model to project the current and future habitat suitability of the pepper weevil under four distinct climate scenarios (SSP1-2.6, SSP2-4.5, SSP3-7.0, and SSP5-8.5) for the periods spanning the 2030s to 2090s. Optimal model performance was achieved with a regularization multiplier of two and a feature combination of QHP, yielding high predictive accuracy with mean testing AUC values of 0.921. The analysis identified annual mean temperature (Bio1) and precipitation of the coldest quarter (Bio19) as the primary environmental factors influencing the pest’s distribution. Currently, in China, suitable habitats for *A. eugenii* encompass an area of 273.74 × 10^4^ km^2^ or 28.47% of the nation’s territory, predominantly located in central, eastern, southern, and southwestern regions. Future projections suggest that suitable areas are expected to shrink across various scenarios, barring increases in specific instances like SSP126-2050s, SSP245-2070s, and SSP370-2050s, with shifts towards southwestern regions. This investigation deepens our comprehension of agricultural pest dynamics under climate change and supports the formulation of preemptive management strategies to safeguard agricultural productivity.

## Simple Summary

The pepper weevil, *Anthonomus eugenii* Cano, stands out as a particularly destructive pest, causing substantial losses in *Capsicum* pepper production. Therefore, accurately identifying its distribution is crucial for effective monitoring and prevention strategies under climate warming scenarios. Using MaxEnt modeling and ArcGIS, this study mapped current and future suitable habitats for the pepper weevil in China under four climate scenarios from 2030 to 2090. The findings indicated that while the pest currently occupies 28.47% of China’s area, future climate changes could lead to reduced suitable habitats, except in certain scenarios, with shifts towards southwestern regions expected. Our findings offer crucial insights for early monitoring and managing of *A. eugenii* populations, as well as for choosing suitable sites for pepper cultivation that are free from pest infestations.

## 1. Introduction

Global climate warming, driven by a combination of natural factors like solar activity and human activities, particularly greenhouse gas emissions [1], significantly affects biodiversity and insect distribution patterns [2]. The Intergovernmental Panel on Climate Change (IPCC) reports that the global temperature has increased by approximately 0.8 °C over the past century and is projected to continue rising due to increasing greenhouse gas emissions [3]. Insects, being ectothermic organisms, experience shorter developmental periods and faster reproductive rates with rising temperatures [4,5,6,7]. Additionally, their overwintering survival rates improve as temperatures increase [8,9,10,11]. An organism is considered a pest when, for example, it threatens plants of economic interest to humans, whereas in natural ecosystems, all organisms play important roles and are not inherently pests. Consequently, climate warming may enhance pest spread and exacerbate crop damage [10], as pest distribution ranges are anticipated to shift towards higher altitudes and latitudes, intensifying agricultural production risks [12,13,14,15,16]. Thus, understanding the impacts of climate change on pest distribution is essential for effective pest management and can offer valuable insights for sustainable crop cultivation practices.

The pepper weevil (PW), *Anthonomus eugenii* Cano, 1894 (Coleoptera: Curculionidae), is considered a significant pest of the cultivated *Capsicum* species [17,18,19]. The pepper weevil is characterized by its dark mahogany to black oval body, which is covered with small whitish setae [18]. It is not only attracted to and capable of reproducing within the fruit of these plants but also within other members of the Solanaceous family such as eggplant (*Solanum melongena* L.) and the common black nightshade (*Solanum americanum* Mill.) [17,18,19,20,21]. The pepper weevil, first described as a pest of peppers in Guanajuato, Mexico by Cano Alcacia in 1894 [18], is likely native to Mexico, according to a recent mitochondrial genome study by van de Vossenberg et al. [22]. *Anthonomus eugenii*, currently found in southern mainland USA, Hawaii, Central America [23], and the Caribbean [24], has also sporadically spread to other parts of the globe including Canada [25], Italy [26], and the Netherlands [27], posing a threat to other major pepper-producing regions in Asia, Europe, and Africa [28]. In the USA, an estimated $23 million in annual crop losses is attributed to *A. eugenii* [29], while a single outbreak of the pest in southern Ontario, Canada, in 2016 caused an estimated $49 million in crop losses [30]. Therefore, the substantial economic impact of pepper weevil infestations and the continued risk that this pest represents on a global scale underscore the importance of understanding its occurrence patterns and implementing targeted preventive measures.

Feeding activity by adult weevils inflicts damage upon fruits, flowers, and buds, while oviposition and larval feeding significantly reduce marketable fruit yields [31]. All immature life stages develop within the confines of these fruits, rendering them impervious to insecticides aimed at controlling adult pepper weevils. This biological characteristic poses a formidable challenge to pest management strategies [17,31]. Presently, the integrated approach combining chemical insecticides, meticulous crop scouting, and cultural management practices constitutes the cornerstone of strategies employed by pepper growers to mitigate pepper weevil populations [29]. Nonetheless, historical accounts of pepper weevil management underscore the inherent difficulties in curbing this pest through chemical means alone, despite their erstwhile prominence as a primary control measure [32,33]. The elusive nature of the pepper weevil’s life stages, concealed within the protective environment of pepper buds and fruits and shielded from insecticidal interventions, coupled with the availability of alternative Solanaceous hosts facilitating re-infestation, intensifies control challenges. Moreover, the negative impacts of insecticide use include environmental contamination, harm to non-target organisms, and potential health risks to humans through exposure to chemical residues [34]. Insecticides can disrupt ecosystems, leading to declines in biodiversity and the development of pesticide-resistant pests. Additionally, improper use and over-reliance on chemicals can result in soil degradation and water pollution [35]. Compounding these issues, the global phase-out of certain insecticide classes, such as neonicotinoids, diminishes the arsenals available to growers for effective pest control. Consequently, should the pepper weevil establish itself in novel pepper production regions or countries, it poses a grave threat to local industries due to the absence of robust prevention and control measures.

China is a global leader in the production and import of *Capsicum* sp. peppers, accounting for nearly half of the world’s fresh chili and bell pepper output [35,36]. This dominance supports a complex network of processed products and industrial chains, significantly contributing to the economy with extensive cultivation areas exceeding 0.76 million hectares and an annual production volume of approximately 16.84 million tons [36]. Although *A. eugenii* has not yet been found in China, the country, as both a major producer and importer, faces heightened risks from pests like the pepper weevil, which has caused substantial economic damage in regions with similar climatic conditions, such as the USA and Canada. The frequent international trade and transportation associated with this commodity increase the likelihood of pest invasion, necessitating proactive measures to prevent the establishment and spread of *A. eugenii* within China’s lucrative pepper sector. Moreover, factors such as climate change and the dynamics of international trade can exacerbate the threat posed by invasive pests, such as the red imported fire ant (*Solenopsis invicta*) (Hymenoptera: Formicidae) [37]. Therefore, understanding potential distribution patterns amidst changing climates is crucial for informing targeted pest management strategies, thereby mitigating risks to this vital agricultural sector and preserving China’s leadership position in global pepper production [22].

Species distribution models (SDMs) are empirical methodologies employed to quantify the ecological niche of a species within its environment. These models infer relationships between species occurrence and environmental variables by integrating data samples of the target species with environmental characteristics from sample locations, thereby predicting potential distribution patterns [38]. Among commonly utilized niche models, such as BIOCLIM, GARP, DOMAIN, and MaxEnt (v.3.4.1) [35], the MaxEnt model stands out for its widespread application across various domains. This popularity stems from its capacity to deliver high-accuracy predictions using small sample sizes, alongside its rapid computation speed and user-friendly operation [39,40]. MaxEnt has been instrumental in assessing climate impacts on species [41] and evaluating invasive species [42,43], among other applications. Notably, MaxEnt has successfully predicted suitable habitat areas for diverse invasive pests, which are non-native organisms that cause ecological or economic harm upon establishment and spread in a new environment. These include *Bactrocera tsuneonis* (Miyake) (Diptera: Tephritidae) [44], *Neoceratitis asiatica* (Becker) (Diptera: Tephritidae) [45], *Paederus fuscipes* Curtis, 1826 (Coleoptera: Staphylinidae), *Spodoptera exempta* (Lepidoptera: Noctuidae) [46], and *Carposina coreana* Kim (Lepidoptera: Carposinidae) [47]. This success underscores its versatility and utility in pest management and ecological assessments.

Currently, research on *A. eugenii* within China remains relatively limited. The extant literature from abroad predominantly focuses on its biological characteristics [28,48,49,50], population ecology [51,52,53], and management strategies [33,54,55,56]. Notably, there is a paucity of studies investigating the potential suitable habitats for this pest species. Consequently, our study employed the optimal MaxEnt model to forecast both the current and future geographical distributions of *A. eugenii* under climate warming scenarios, while also identifying the primary environmental determinants shaping its distribution patterns. This research aimed to furnish valuable insights for enhancing pest monitoring and management practices and for pinpointing pest-free zones conducive to pepper cultivation.

## 2. Materials and Methods

### 2.1. Collection and Screening of Species Geographic Distribution Points

**Figure 1:**
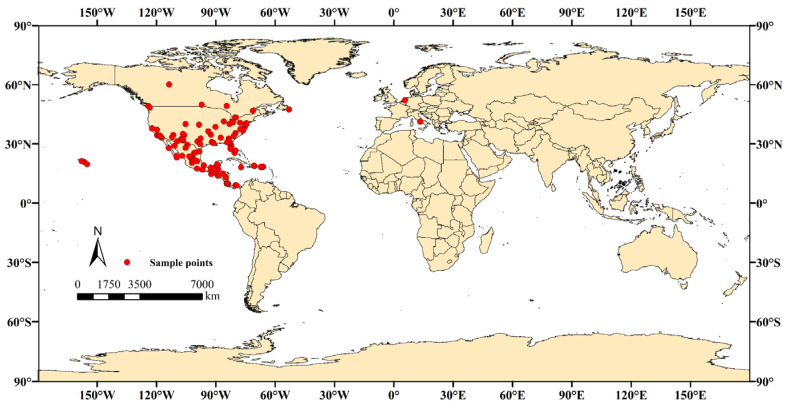
Worldwide distribution map of *A. eugenii* incidences.

This study meticulously compiled a dataset comprising 148 occurrence records of *A. eugenii* (Figure 1), sourced from an array of authoritative repositories, including the Global Biodiversity Information Facility (GBIF: https://www.gbif.org, accessed on 8 September 2024), CABI International Centre for Applied Biological Sciences’ PlantwisePlus (https://plantwiseplusknowledgebank.org/, accessed on 8 September 2024), the “National Directory of Agricultural Plant Quarantine Harmful Organisms Distribution by Administrative Region” (https://www.moa.gov.cn/nybgb/, accessed on 3 September 2024), Bold Systems v4 (http://www.boldsystems.org/, accessed on 5 September 2024), and iNaturalist (https://www.inaturalist.org/, accessed on 5 September 2024), along with additional data retrieved from scholarly literature indexed within the CNKI and Web of Science databases. Recognizing the potential biases inherent in occurrence records that could compromise the accuracy of predictive models, a rigorous two-stage filtering protocol was implemented. Initially, records lacking sufficient detail were systematically excluded to mitigate the impact of data incompleteness. Subsequently, the remaining dataset underwent purification using ENMtools v1.4 (https://www.example.com/enmtools, accessed on 20 September 2024), ensuring spatial uniqueness at a resolution of 2.5 arcminutes per raster cell. This refined approach yielded a final selection of 124 high-quality occurrence records, deemed suitable for deployment in the MaxEnt modeling framework (Figure S1). For an exhaustive compilation of these distribution points, along with corresponding cartographic representations, refer to Figure S1 provided in the Supplementary Materials section.

### 2.2. Environmental Variables and Selection Criteria

The environmental variables employed in this study were sourced exclusively from the Worldclim database (www.worldclim.org, accessed on 24 May 2023), characterized by a spatial resolution of 2.5 arcmin and utilizing version 2.1 of the dataset [57]. Historical climate data spanning the period from 1971 to 2000 were selected for the analysis of environmental variables, while future climate projections encompassed four distinct time intervals: 2021–2040, 2041–2060, 2061–2080, and 2081–2100 [58]. These future climate scenarios were derived from the Beijing Climate Center Climate System Model 2 Medium Resolution (BCC-CSM2-MR) within the framework of the 6th International Coupled Model Intercomparison Project Phase 6 (CMIP6) [59], featuring four shared socioeconomic pathways: SSP1-2.6, SSP2-4.5, SSP3-7.0, and SSP5-8.5. These pathways represent diverse socio-economic development trajectories tailored to current national and regional contexts and strategic plans, with SSP1-2.6 categorized under low forcing, SSP2-4.5 under medium forcing, and both SSP3-7.0 and SSP5-8.5 falling within the high forcing category. Each scenario postulates a stabilization of radiative forcing by the year 2100 at levels of 2.6, 4.5, 7.0, and 8.5 W/m^−2^, respectively [60].

**Figure 2:**
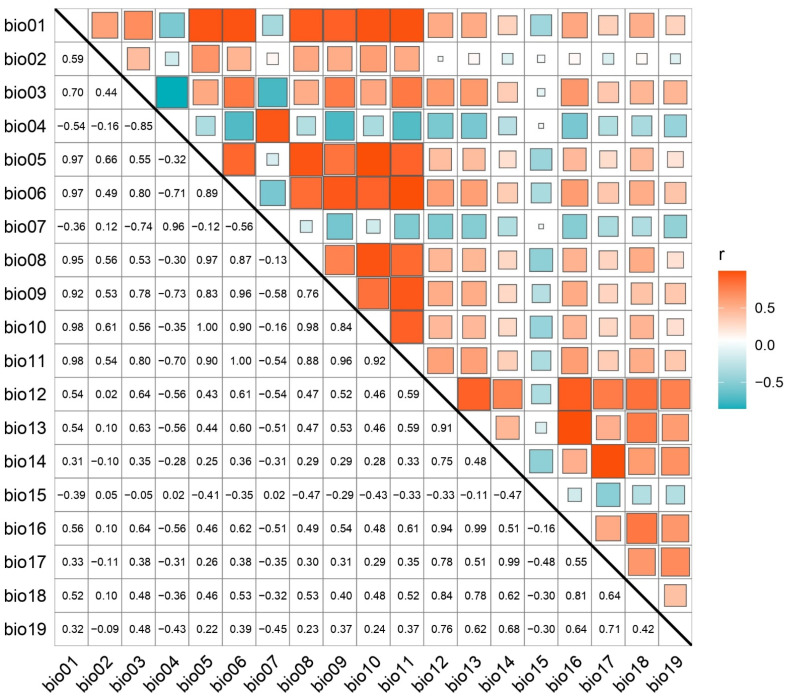
Pearson correlation coefficients for 19 environmental variables (positive correlations in red, negative in blue).

In forecasting species distribution, accurately identifying key environmental factors that significantly influence habitat suitability is paramount. Consequently, a stringent variable selection methodology was implemented to eliminate those with negligible impact on species distribution patterns. Initially, occurrence records of *A. eugenii* alongside nineteen bioclimatic variables were integrated into MaxEnt software to establish a preliminary model, configured with a random test percentage of 25%. This was followed by a jackknife test to evaluate each variable’s individual contribution and permutation significance to the model’s initial outcomes. To mitigate spatial autocorrelation among variables, the collected distribution data facilitated the extraction of values for nineteen environmental layers using ArcGIS 10.4.1. Subsequent Pearson correlation analyses conducted in R software (v.4.2.3) identified variables exhibiting correlation coefficients exceeding |0.8| as highly correlated and thus redundant (Figure 2). From each pair of such variables, one was retained based on its relative importance in modeling *A. eugenii*’s potential distribution, thereby distilling the principal environmental determinants for the final MaxEnt model, which incorporated five bioclimatic variables (Table S1).

### 2.3. MaxEnt Model Optimization

The employment of default parameters in the construction of a MaxEnt model can lead to overfitting, where the model’s predictions become excessively tailored to the specific training data [61]. To mitigate this issue, the ENMeval package within R software was utilized to adjust two critical parameters: the feature combination (FC) and the regularization multiplier (RM) of MaxEnt [62]. MaxEnt offers five distinct features for ecological niche modeling: linear (L), quadratic (Q), hinge (H), product (P), and threshold (T). In this study, RM values ranged from 0.5 to 4, with increments of 0.5, and eight different feature combinations were tested: L, LQ, LQP, QHP, LQH, LQHP, QHPT, and LQHPT [63]. The suitability and complexity of the model were evaluated using the corrected Akaike information criterion (AICc) [64,65], while the extent of overfitting was assessed through a 10% training omission rate (OR_10_) [66]. The optimization process leveraged the capabilities of the ENMeval package in R, aiming to identify the optimal model configuration based on achieving the lowest AICc value, which indicates a good balance between model fit and predictive accuracy.

**Figure 3:**
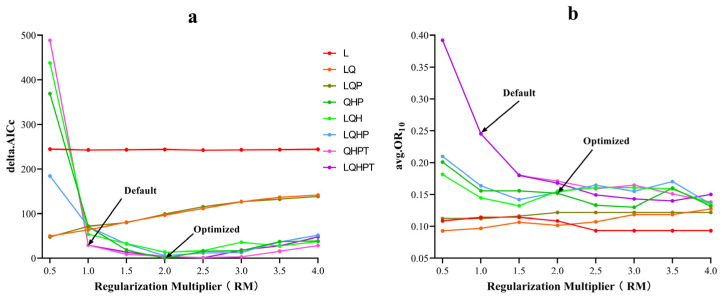
Evaluating MaxEnt model performance across different parameters. (**a**) delta.AICc values; (**b**) OR_10_ Values. Legend for feature classes: L = linear, Q = quadratic, H = hinge, P = product, T = threshold.

The default settings for the Maxent model were RM = 1, FC = LQPHT, and a delta.AICc of 29.23. However, Figure 3 revealed that the lowest AICc value (delta.AICc = 0) was achieved with RM = 2 and FC = QHP. This parameter combination also resulted in the lowest model complexity according to the Akaike information criterion. Furthermore, the mean OR_10_ value was 15.19% lower with this optimized setup compared to the default parameters (Figure 3). After 10 iterations, the optimized Maxent model exhibited a mean testing AUC value of 0.921 (Figure S2), indicating improved model fit, flexibility, and reduced overfitting.

### 2.4. MaxEnt Model Evaluation and Suitable Habitats Prediction

The 128 occurrence records of *A. eugenii* and five bioclimatic variables were incorporated into the MaxEnt model. Seventy-five percent of these occurrence records were randomly designated as the training set, while the remaining 25% served as the testing set. The parameters for the MaxEnt model were configured based on the optimized feature combination. Additionally, the background points were set to 10,000, the maximum iterations were capped at 5000, the output format was specified as logistic, and the model runs were repeated 10 times for cross-validation purposes. The jackknife method was employed to test and generate response curves, which helped in evaluating the impact of bioclimatic variables on the potential suitable habitat area for *A. eugenii* in China, with the model’s accuracy assessed using the area under the receiver operating characteristic (ROC) curve (AUC) [67]. The AUC values ranged from 0 to 1, with higher values indicating superior model prediction accuracy: below 0.5 signifies a failed prediction, 0.6–0.7 indicates poor performance, 0.7–0.8 is considered usable, 0.8–0.9 is good, and above 0.9 is excellent.

In this study, the final results, comprising average values from 10 repetitions of the MaxEnt model, delineated the potential suitable habitat areas for *A. eugenii* in China. These results were obtained by evaluating the presence probability of *A. eugenii*, with values ranging from 0 to 1, where higher values indicated a greater likelihood of species presence. The model outputs were imported into ArcGIS software using the reclassify tool, which classified the potential suitable areas for *A. eugenii* into four categories based on natural breaks (Jenks) method: 0–0.097 was classified as an unsuitable area, 0.098–0.28 as a poorly suitable area, 0.29–0.51 as a moderately suitable area, and 0.52–0.95 as a highly suitable area.

### 2.5. Centroid Shifts in Suitable Habitats

The centroid, a valuable metric for characterizing the spatial distribution of geographical entities and monitoring their displacement over time [44,45,46], was examined in this study to assess the shifts of *A. eugenii* within nationally suitable habitats under anticipated future climate scenarios. Initially, the habitat raster map was transformed into a vector format utilizing ArcGIS software. Subsequently, analysis was conducted by incorporating a folder containing both current and future binary species distribution models (SDMs) into the SDMtoolboxw2.5 tool (http://www.sdmtoolbox.org/downloads, assessed on 27 September 2024) [68].

## 3. Results

### 3.1. Key Bioclimate Variables and Response Curves

Table 1 presents the percentage contributions and permutation importance values for five bioclimatic variables. Among these, annual mean temperature (Bio1) emerged as the most significant factor, boasting a contribution rate of 52.2% and a permutation importance of 43.1%. This underscores Bio1’s pivotal role as the primary determinant of temperature, which in turn influences the distribution of *A. eugenii*. Furthermore, the contributions from other key variables were also notable: precipitation of coldest quarter (Bio19) at 27.6%, mean diurnal temperature range (Bio2) at 11.3%, and precipitation of warmest month (Bio13) at 8%.

**Figure 4:**
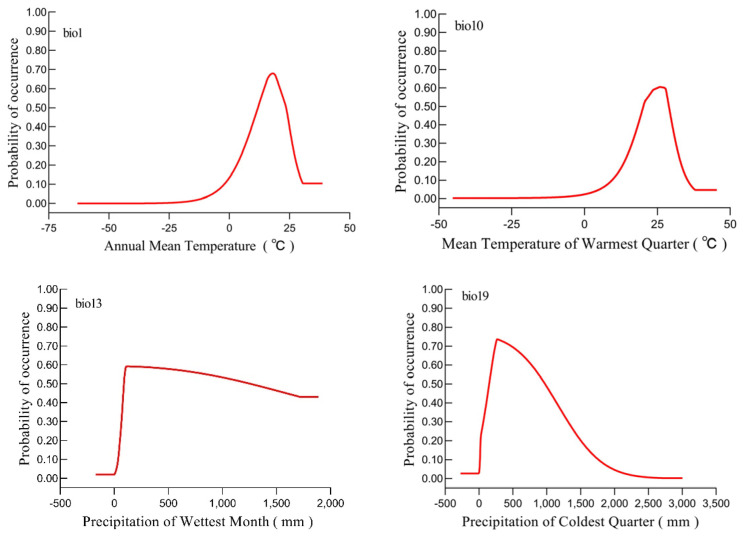
Probability distribution of *A. eugenii* across varied bioclimatic variables.

The individual response curves for each bioclimatic variable were presented in Figure 4. For *A. eugenii*, the suitable ranges of values for these variables (with a probability ≥ 0.29) were as follows: 6.01–26.53 °C for Bio1, 58–1338.45 mm for Bio19, 65.28–1889 mm for Bio13, and 6.10–31.54 °C for Bio10. In terms of temperature, both Bio1 and Bio10 exhibited similar patterns: within a certain range, the probability of *A. eugenii* occurrence increased with increasing Bio1 and Bio10 values. After reaching a peak, the probability decreased with further increases in these environmental factors. The optimal temperatures for *A. eugenii* occurrence were 17.75 °C and 25.91 °C for Bio1 and Bio10, respectively, with corresponding output probabilities of 67.93% and 60.52%. When modeling habitat suitability in new regions, we have opted to exclude Bio2 from our analysis due to its lack of a closed, bell-shaped response curve, which is desirable for accurately projecting the model in areas where the species has not yet been established. Regarding precipitation, when Bio19 and Bio13 exceeded 0, the presence probability of *A. eugenii* sharply increased, peaking at 271.41 and 116.82, with the probability reaching as high as 73.64% and 59.26%, respectively. Subsequently, the Bio19 curve declined sharply while the Bio13 curve decreased more smoothly.

### 3.2. Current Distribution Prediction

**Figure 5:**
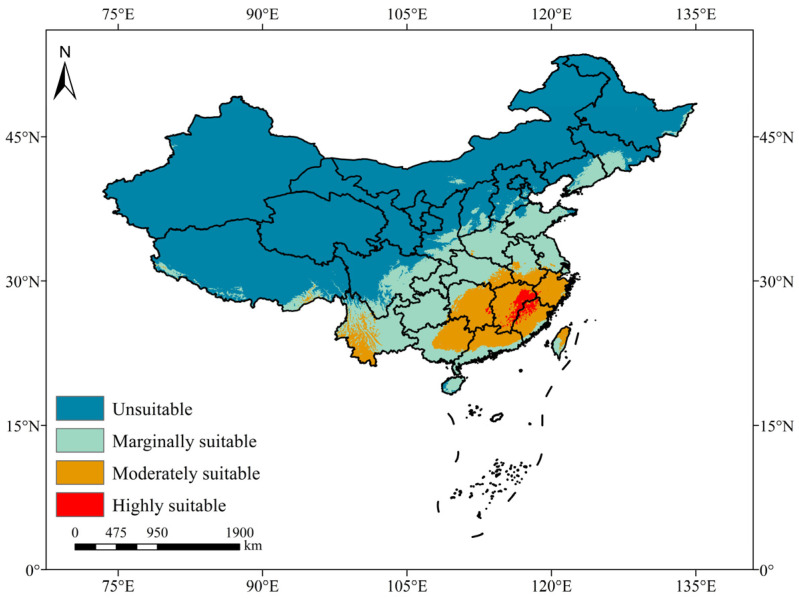
Map of potentially suitable areas for *A. eugenii* under present climatic conditions in China.

The present distribution of *A. eugenii* was charted based on simulation outcomes (Figure 5). Under current climatic conditions, the total estimated suitable distribution area spanned approximately 273.74 × 10^4^ km^2^, constituting 28.47% of the total national territory, mainly concentrated in central, eastern, southern, and southwestern China. Among these, regions categorized as marginally, moderately, and highly suitable covered approximately 184.63 × 10^4^, 83.43 × 10^4^, and 5.68 × 10^4^ km^2^, respectively, accounting for 67.44%, 30.48%, and 2.08% of the overall suitable area. Notably, areas classified as highly suitable were predominantly concentrated in the southeast of Jiangxi, the north of Fujian, and sporadic portions of Hunan and Zhejiang. Interestingly, the boundary between the low-suitability and non-suitability areas for the pepper weevil was basically distributed along the 800 mm isohyet.

**Figure 6:**
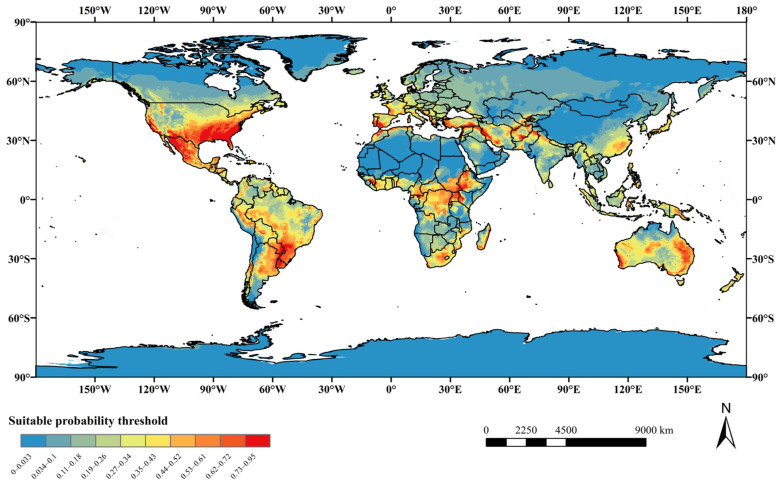
Map of potentially suitable areas for *A. eugenii* globally under present climatic conditions.

As shown in Figure 6, the simulation results for the occurrence records of *A. eugenii* indicate its current predicted distribution area, with varying suitability grades distinguished by distinct colors representing high, moderate, and marginal suitability. The results showed that the suitable areas for *A. eugenii* were mainly distributed between 50° N and 50° S, including the southern regions of North America, most of South America, the central and southern coasts of Africa, Europe, South Asia, West Asia, Southeast Asia, and Oceania.

### 3.3. Future Distribution Prediction

**Figure 7:**
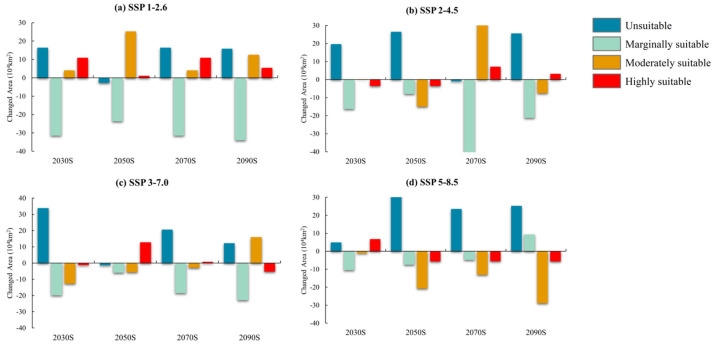
Changes in suitable habitat areas from current to future periods under climate change models in China.

**Figure 8:**
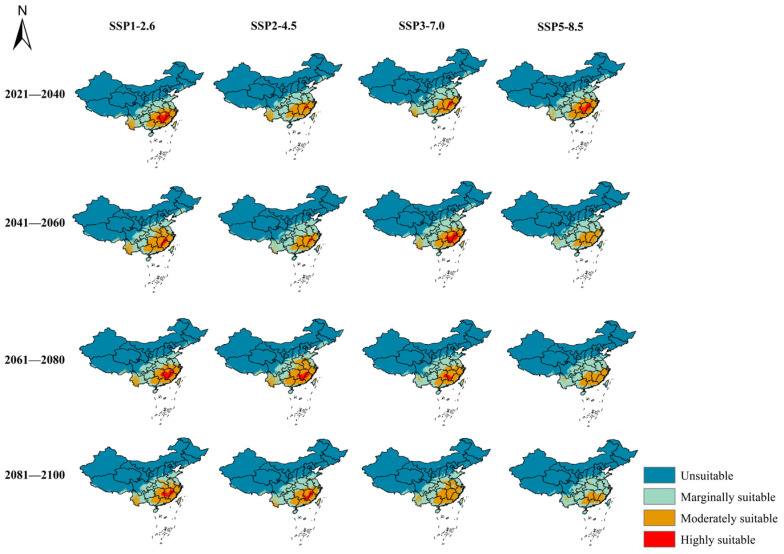
Habitat suitability of *A. eugenii* under diverse climate change projections in China.

The potential distribution of *A. eugenii* under four emission scenarios (SSP1-2.6, SSP2-4.5, SSP3-7.0, and SSP5-8.5) across four future periods (2030s, 2050s, 2070s, and 2090s) is illustrated in Figure 7 and Figure 8, with details provided in Table 2. Overall, the total suitable area for *A. eugenii* was projected to decrease to varying extents under future climate scenarios compared to current conditions, except during the 2050s under SSP1-2.6 and SSP3-7.0 and the 2070s under SSP2-4.5. Under future climate scenarios, the suitable habitat for *A. eugenii* in China will consistently remain within these provinces and regions such as Fujian, Jiangxi, Zhejiang, Shanghai, Jiangsu, Guangdong, Guangxi, Chongqing, Guizhou, Yunnan, Hubei, Hunan, Henan, Anhui, Zhejiang, Hainan, Taiwan, etc. The habitats in these regions exhibit varying degrees of suitability, including high, medium, and marginal levels. However, the specific degree of suitability may change under different climate models.

Under the SSP1-2.6 scenario, both highly and moderately suitable habitats for the pepper weevil expanded to varying degrees across different periods compared to the current period, while the low suitability habitat significantly contracted. Specifically, the highly suitable habitat expanded by 10.92% in the 2030s, 1.15% in the 2050s, 10.92% in the 2070s, and 5.4% in the 2090s. In contrast, under the SSP2-4.5 scenario, the area of the highly suitable habitat shrank during the 2030s and 2050s but expanded during the 2070s and 2090s. The moderately suitable habitat saw expansion during the 2030s and 2070s, with a notable increase of 34.32% in the 2070s. However, the low suitability habitat contracted across all periods, and the unsuitable habitat area significantly expanded, with the only exception being a slight contraction during the 2070s.

For the SSP3-7.0 scenario, the highly suitable habitat for the pepper weevil generally contracted in most periods, with significant expansions observed only in the 2050s and minor expansion in the 2070s. The moderately suitable habitat experienced a notable expansion in the 2090s, with an expansion rate of 15.95%, although it contracted compared to the current period in other periods. Similarly, the low suitability habitat also contracted compared to the current period. Under the SSP5-8.5 scenario, except for an expansion during the 2030s, the area of the highly suitable habitat contracted in other periods, and by the 2090s, there were no regions in China that remained highly suitable for the pepper weevil. Both the moderately suitable and low suitability habitats contracted across all periods, except for a slight increase in the low suitability habitat in the 2090s. Additionally, the area of the unsuitable habitat expanded throughout.

### 3.4. Centroid Change in the Suitable Habitats of A. eugenii

**Figure 9:**
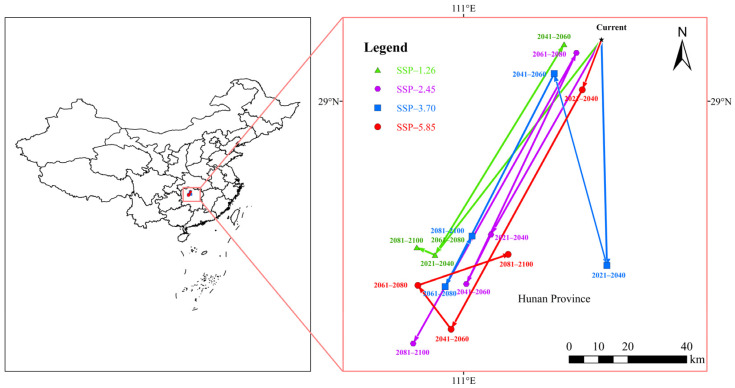
Shifts in geographical centers of *A. eugenii*’s potential distribution under current and future climate scenarios.

Figure 9 and Table 3 illustrate the centroid displacement trajectories of suitable habitats for *A. eugenii* under four different climate change scenarios over the next four periods (2021–2040, 2041–2060, 2061–2080, and 2081–2100). The current period’s centroid was located in Changde City, Hunan Province (111.421° E, 29.188° N), and under future climate scenarios, the centroids also moved within Hunan Province. Under future climate scenarios, there were minimal changes in the overall spatial distribution of suitable habitats for the pepper weevil, with an overall migration towards the southwest direction.

## 4. Discussion

### 4.1. Dominant Bioclimatic Variables Influencing the Suitable Areas of A. eugenii

Climate, including temperature and precipitation, is a primary factor shaping species distribution [69] and directly influences the life activities of insects such as growth, reproduction, survival, and interactions with host plants. The distribution of *A. eugenii* is significantly influenced by two key environmental variables: annual mean temperature (Bio1) and precipitation of the coldest quarter (Bio19). Temperature has the most significant impact on the distribution of *A. eugenii*, followed by precipitation, with Bio1 (annual mean temperature) being the most influential factor, having a suitable range of 6.01–26.53 °C. Previous research meticulously characterized the developmental biology and life history parameters of *A. eugenii* across a range of constant temperatures (15 to 33 °C) in a controlled laboratory setting. This rigorous experimental design allows for precise measurement of how temperature influences various aspects of the weevil’s biology, including its developmental threshold and degree-day requirements. The finding that 9.6 °C is the lower developmental threshold from egg, this aligns well with the identified suitable annual mean temperature range (6.01–26.53 °C) for *A. eugenii* distribution [70]. Furthermore, considering that *A. eugenii* is a major pest of peppers in tropical and subtropical America, where temperatures generally fall within the identified suitable range, this real-world observation lends ecological validity to the prediction findings. It implies that under natural conditions, areas with annual mean temperatures within 6.01–26.53 °C provide the necessary thermal environment for *A. eugenii* to thrive, thereby supporting our conclusion about the primary influencing factor on its distribution.

Furthermore, Fernández [71] investigated the cold tolerance of the pepper weevil through both field and laboratory experiments. An acclimation process designed to mimic the gradual onset of winter conditions was applied to some individuals in an effort to enhance their cold tolerance. Despite these efforts, neither adults nor larvae survived freezing temperatures or even temperatures above the supercooling point during assessments. His findings indicated that the pepper weevil is highly susceptible to chilling, and below zero temperatures will restrict the establishment of pepper weevil outdoors, as it lacks effective strategies for surviving cold conditions. These findings align with our prediction that the lower limit of the suitable temperature range for the pepper weevil (6.01 °C) is higher than its supercooling point. These studies collectively demonstrated that temperature is a critical environmental variable influencing the distribution of *A. eugenii*.

The study’s findings confirmed that precipitation, which has both direct and indirect effects on crop insect pests [72], is another key environmental factor influencing the distribution of *A. eugenii*. In recent years, environmental shifts attributed to global warming have led to alterations in moisture content and rainfall patterns, directly impacting insect dynamics. China, influenced by the East Asian monsoons and western circulation, experiences seasonal and regional variations in dryness and precipitation [73]. Additionally, environmental moisture profoundly affects the timing, abundance, and geographical range of forestry and agricultural pests [74]. This highlights the significance of Bio19 (precipitation of the coldest quarter) and Bio13 (precipitation of the wettest month) in influencing the distribution of *A. eugenii*, with the greatest effect being Bio19, where the suitable range for this variable was found to be 58–1338.45 mm. Currently, there is a lack of reports on the direct impact of environmental factors such as precipitation and humidity on the pepper weevil. The life cycle of the pepper weevil is predominantly spent on its host plant. Female adult pepper weevils lay eggs inside flower buds or fruits, subsequently sealing the borehole with a viscous fluid. Once hatched, larvae immediately begin feeding on the soft tissues within the fruit. Third-instar mature larvae pupate internally, and upon reaching adulthood, remain within the fruit until they are sexually mature and then bore their way out [31]. Consequently, variations in precipitation may influence the growth of the host plants, which in turn affects the development and growth of the pepper weevil. Previous studies have confirmed that winter precipitation below 500 mm is favorable for the growth of pepper in northern Guizhou Province, China [75], which coincides with the projected precipitation range required for the suitable habitat of *A. eugenii.* The results of this study indicated that Guizhou Province, which remains the largest province in China in terms of pepper planting area and output, is still considered potentially suitable for the proliferation of *A. eugenii* under current conditions and projected future climate scenarios. Over the past 40 years, the annual precipitation recorded in Guizhou ranged from 856 to 1318 mm [76]. The anticipated precipitation threshold for the suitability of *A. eugenii* in our study was consistent with the historical annual precipitation range in this area, confirming the robustness of our findings.

### 4.2. Alterations in Suitable Habitats of A. eugenii

The prediction results of the MaxEnt model under the current scenario indicated that, with the exception of Antarctica, all continents possessed regions suitable for the growth of *A. eugenii*. A comparison between the predicted suitable distribution areas and the actual occurrence points revealed a high degree of correspondence in North America, suggesting that the predicted suitable habitats closely align with known distribution sites. This finding underscored the strong credibility of the MaxEnt model we developed for forecasting suitable regions. Moreover, suitable habitats for *A. eugenii* in China under current climatic conditions are primarily located in provinces or regions such as Fujian, Jiangxi, Zhejiang, Shanghai, Jiangsu, Guangdong, Guangxi, Chongqing, Guizhou, Yunnan, Hubei, Hunan, Henan, Anhui, Zhejiang, Hainan, Taiwan, and Sichuan. The habitats in these regions exhibit varying degrees of suitability, including high, medium, and marginal suitability. All the provinces predicted to be suitable for pepper weevil habitats are also important regions for pepper production in China, each with its unique varieties and characteristics, collectively forming the diverse pepper culture of China. Furthermore, under several future climate model projections, these provinces and regions will remain suitable habitats for the pepper weevil, albeit with varying degrees of suitability. For example, under the SSP5-8.5 scenario, Fujian Province will not have highly suitable habitats in the 2070s and 2090s, shifting instead to moderate or low suitability levels. The three provinces with a national pepper planting area exceeding 200,000 hectares, namely Guizhou, Henan, and Hunan, are all suitable areas in our current and future predictions (Figure S3) [77]. Therefore, it is imperative to exercise caution in preventing the invasion and spread of *A. eugenii* into these regions by emphasizing biosecurity surveillance as a crucial element in the early detection of invasive alien species [78]. The pepper weevil exhibits a significant attraction to male-produced aggregation pheromones and herbivore-induced plant volatiles (HIPVs) [79], which can be further explored and integrated with trapping mechanisms for the early detection of pepper weevils. Additionally, relevant departments should establish comprehensive monitoring and evaluation protocols for plant products, derivatives, and by-products associated with the host of *A. eugenii* to prevent its invasion and diffusion within China.

Under global climate warming scenarios, the severity of pest infestations is expected to increase as ambient temperatures approach optimal levels for pest development, potentially alleviating thermal constraints on population dynamics [10]. However, our results indicate a reduction in the suitable area for *A. eugenii* under future climate scenarios, ranging from 1.79% to 12.38%, with exceptions for SSP126-2050s, SSP 245–2070s, and SSP 370–2050s, possibly due to the pest’s narrow niche requirements and poor physiological tolerance. Lehmann et al. [80] observed that climate change will alter the potential distribution of insect pests in a species-specific manner, with various pests showing diverse responses to climate warming. Specifically, 59% of these pests are expected to mitigate their detrimental impact primarily through reduced physiological performance and contraction in geographic range.

Climate change is widely acknowledged to drive species migration toward higher latitudes and altitudes, with more pronounced effects observed in high-altitude regions due to significant temperature increases compared to lower elevations [81]. The adaptability of insect pests to rising temperatures and fluctuating rainfall plays a crucial role in determining their future distribution. Our research indicated that the suitable habitat for *A. eugenii* had shifted predominantly southwest under climate warming conditions, aligning with its host, the chili pepper. Deng et al. [36] projected that by the 2050s, the centroids of suitable habitats for the chili pepper would shift southwest from their current locations in China. This synchronization between pest and host highlights the critical need for future prevention and monitoring efforts in pepper-production areas across the country.

### 4.3. Limitations of This Study

The MaxEnt model, known for its limited sample size requirements, short run time, ease of use, and high simulation precision, was employed in this study to predict the distribution probability of the target species based on known distribution data and selected environmental factors. However, despite its good predictive performance, the model has limitations due to the restricted number of environmental variables considered; only temperature and precipitation were included, while other influential factors such as altitude, host range, cultivation type, vegetation type, human activities, natural enemies, and the insects’ capacity for adaptation and evolution were not incorporated, inevitably leading to biases in prediction results. Additionally, the response curves generated by the model illustrate only the impact of individual environmental factors, disregarding potential interactions between these variables. Given the complexity and multitude of factors influencing insect distributions, it is impractical to comprehensively consider all environmental factors in a single model analysis. Therefore, treating the MaxEnt model as a fundamental niche model may be more effective. Furthermore, the environmental data used for prediction were confined to the period from 1970 to 2000, failing to encompass significant climate changes observed over the past two decades due to heightened greenhouse gas emissions. This lack of contemporary data may have impacted the predictive accuracy of this study. Future research should aim to refine the model by incorporating a more intricate representation of the interplay among various factors to enhance its predictive efficacy. The generated maps serve as indicators of the potential future invasion of *A. eugenii* in China, highlighting the urgent need for additional research on this economically significant agricultural pest, particularly in the development of an integrative pest management system.

## 5. Conclusions

This study utilized the MaxEnt model to effectively predict the potential geographical spread of *A. eugenii* under present conditions and future projections (2030s, 2050s, 2070s, and 2090s) across four distinct climate change scenarios (SSP1-2.6, SSP2-4.5, SSP3-7.0, and SSP5-8.5). The research identified key bioclimatic variables influencing the habitat suitability of *A. eugenii*, with Bio1 (annual mean temperature) and Bio19 (precipitation of the coldest quarter) being the most significant parameters. Currently, the primary habitats of *A. eugenii* are located in eastern, central, southern, and southwestern China, with highly suitable areas concentrated in Fujian and Jiangxi Provinces. Our findings indicate that the area of suitable habitat for this weevil pest is expected to decrease and shift towards the southwest under future climate scenarios compared to current conditions. This study offers new insights into the distribution and environmental impact factors of *A. eugenii* and provides valuable references for its application in agricultural pest control.

## Figures and Tables

**Table 1:** Percent contribution and permutation importance of five core bioclimatic factors.

Variables	Percent Contribution (%)	Permutation Importance (%)
Bio1	52.2	43.1
Bio19	27.6	27.3
Bio2	11.3	11.8
Bio13	8	7.3
Bio10	0.8	10.6

**Table 2:** Variations in suitable habitat areas for *A. eugenii* under diverse climate scenarios.

Scenario	Decade	Predicted Area (10^4^ km^2^)			Comparison with Current Distribution (%)		
		High Suitable	Medium Suitable	Low Suitable	High Suitable	Medium Suitable	Low Suitable
-	Current	5.68	83.43	184.63	——	——	——
SSP1-2.6	2030s	16.60	87.56	153.18	192.30%	4.94%	−17.04%
	2050s	6.83	108.69	160.95	20.29%	30.28%	−12.83%
	2070s	16.60	87.56	153.18	192.30%	4.94%	−17.04%
	2090s	11.15	96.03	150.69	96.33%	15.10%	−18.38%
SSP2-4.5	2030s	2.17	83.65	168.29	−61.83%	0.27%	−8.85%
	2050s	2.16	68.49	176.65	−62.01%	−17.90%	−4.32%
	2070s	12.88	117.75	144.09	126.68%	41.13%	−21.96%
	2090s	8.90	75.81	163.44	56.60%	−9.13%	−11.48%
SSP3-7.0	2030s	4.47	70.72	164.73	−21.24%	−15.23%	−10.78%
	2050s	18.53	77.84	178.72	226.22%	−6.70%	−3.20%
	2070s	6.50	80.58	166.07	14.49%	−3.41%	−10.05%
	2090s	0.35	99.38	161.75	−93.92%	19.12%	−12.40%
SSP5-8.5	2030s	12.41	82.25	174.18	118.43%	−1.41%	−5.66%
	2050s	0.01	62.73	177.10	−99.76%	−24.81%	−4.08%
	2070s	0.05	70.33	179.84	−99.05%	−15.71%	−2.60%
	2090s	0.00	54.56	193.95	−100.00%	−34.60%	5.05%

“——”: Not applicable.

**Table 3:** Centroid displacement trajectory of *A. eugenii* suitable habitats under climate change scenarios.

Current Centroid Location	Climate Scenario	Future Centroid Location			
		2030s	2050s	2070s	2090s
Changde City, Hunan Province (111.421° E, 29.188° N)	SSP1-2.6	Huaihua City (110.912° E, 28.528° N)	Changde City (111.308° E, 29.172° N)	Huaihua City (110.912° E, 28.528° N)	Huaihua City (110.857° E, 28.552° N)
	SSP2-4.5	Changde City (111.085° E, 28.593° N)	Yiyang City (111.008° E, 28.44° N)	Changde City (111.345° E, 29.147° N)	Yiyang City (110.846° E, 28. 26° N)
	SSP3-7.0	Yiyang City (111.438° E, 28.498° N)	Changde City (111.277°E, 29.084°N)	Changde City (110.943° E, 28.433° N)	Huaihua City (111.026° E, 28.588° N)
	SSP5-8.5	Changde City (111.363° E, 29.036° N)	Yiyang City (110.962° E, 28.302° N)	Huaihua City (110.86° E, 28.437° N)	Changde City (111.137° E, 28.532° N)

## Data Availability

The raw data supporting the conclusions of this article will be made available by the authors on request.

## Supplementary Materials

The following supporting information can be downloaded at: https://www.mdpi.com/article/10.3390/insects16020227/s1, Table S1: Set of bioclimatic variables considered for *A. eugenii* habitat modeling in China (Key variables highlighted in bold); Figure S1: Dataset for *A. eugenii* Occurrence Used in MaxEnt Analysis; Figure S2: Assessing MaxEnt Model Performance via Receiver Operating Characteristic (ROC) Curve; Figure S3: Distribution Map of Pepper Planting Areas in China.
