# Supplementary material for: Projecting Current and Future Habitat Suitability of the Pepper Weevil, Anthonomus eugenii Cano, 1894 (Coleoptera: Curculionidae), in China: Implications for the Pepper Industry
Source: Insects. 2025 Feb 19;16(2):227. doi: 10.3390/insects16020227 (PMC11856192; doi:10.3390/insects16020227)
Supplement: Supplementary file 1 [file insects-16-00227-s001.zip › insects-3412457-supplementary.pdf]

Table S1. Set of bioclimatic variables considered for *A. eugenii* habitat modeling in China (Key variables highlighted in bold)

Variables	Description	Unit
**Bio1**	**Annual Mean Temperature**	**°C**
**Bio2**	**Mean Diurnal Temperature Range**	**°C**
Bio3	Isothermality (Bio2/Bio7) (×100)	%
Bio4	Temperature Seasonality (standard deviation ×100)	°C
Bio5	Maximum Temperature of Warmest Month	°C
Bio6	Minimum Temperature of Coldest Month	°C
Bio7	Temperature Annual Range (Bio5-Bio6)	°C
Bio8	Mean Temperature of Wettest Quarter	°C
Bio9	Mean Temperature of Driest Quarter	°C
**Bio10**	**Mean Temperature of Warmest Quarter**	**°C**
Bio11	Mean Temperature of Coldest Quarter	°C
Bio12	Annual Precipitation	mm
**Bio13**	**Precipitation of Wettest Month**	**mm**
Bio14	Precipitation of Driest Month	mm
Bio15	Precipitation Seasonality (Coefficient of Variation)	1
Bio16	Precipitation of Wettest Quarter	mm
Bio17	Precipitation of Driest Quarter	mm
Bio18	Precipitation of Warmest Quarter	mm
**Bio19**	**Precipitation of Coldest Quarter**	**mm**

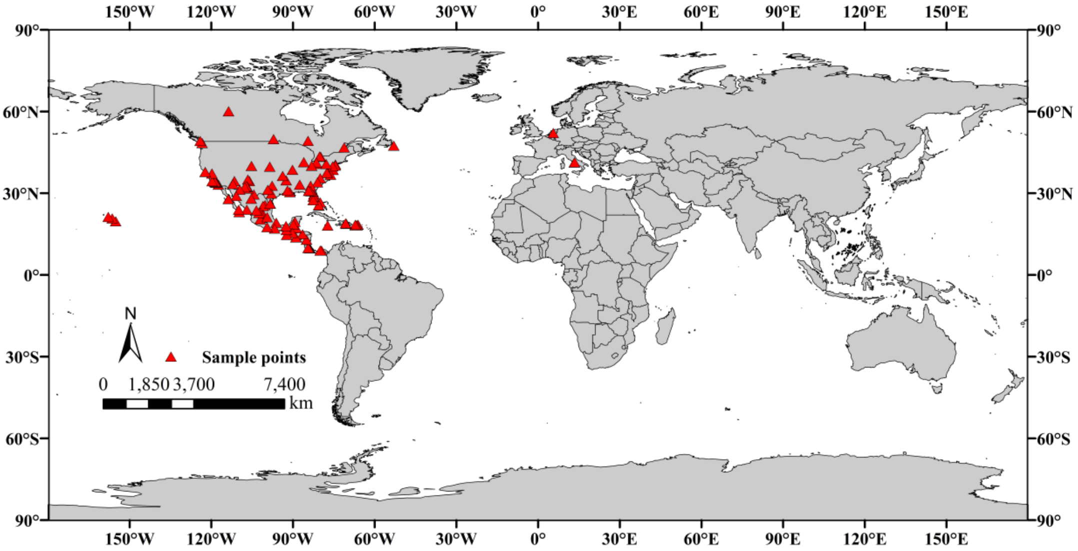

Figure S1: Dataset for *A. eugenii* Occurrence Used in MaxEnt Analysis

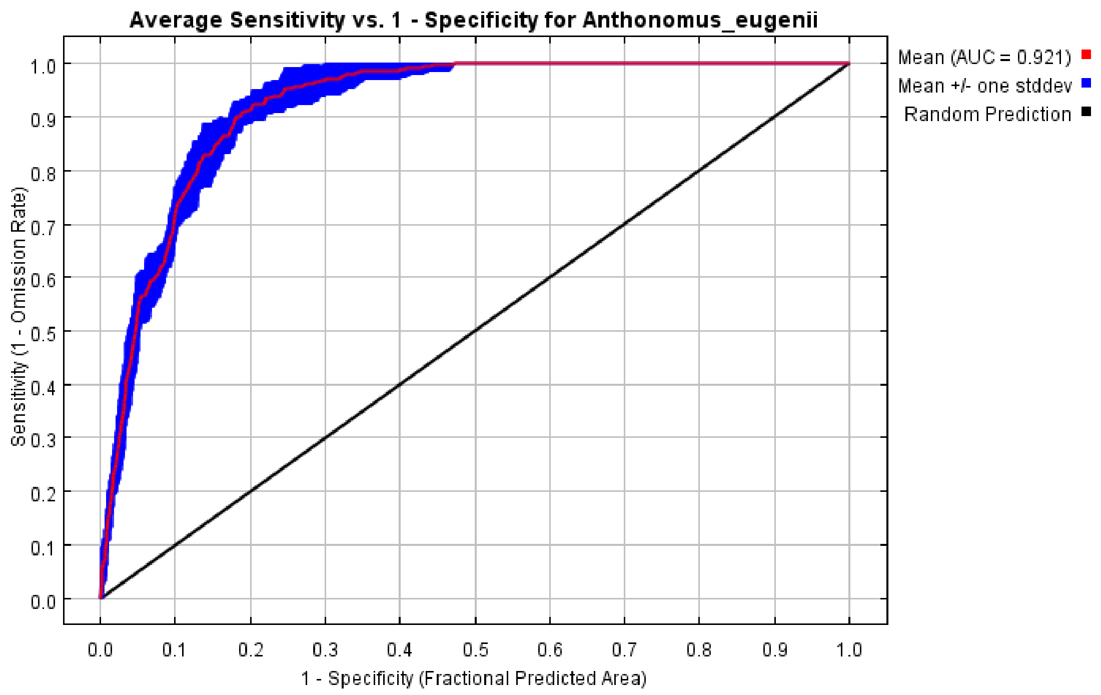

Figure S2 Assessing MaxEnt Model Performance via Receiver Operating Characteristic (ROC) Curve. Assessing MaxEnt Model Performance via Receiver Operating Characteristic (ROC) Curve.

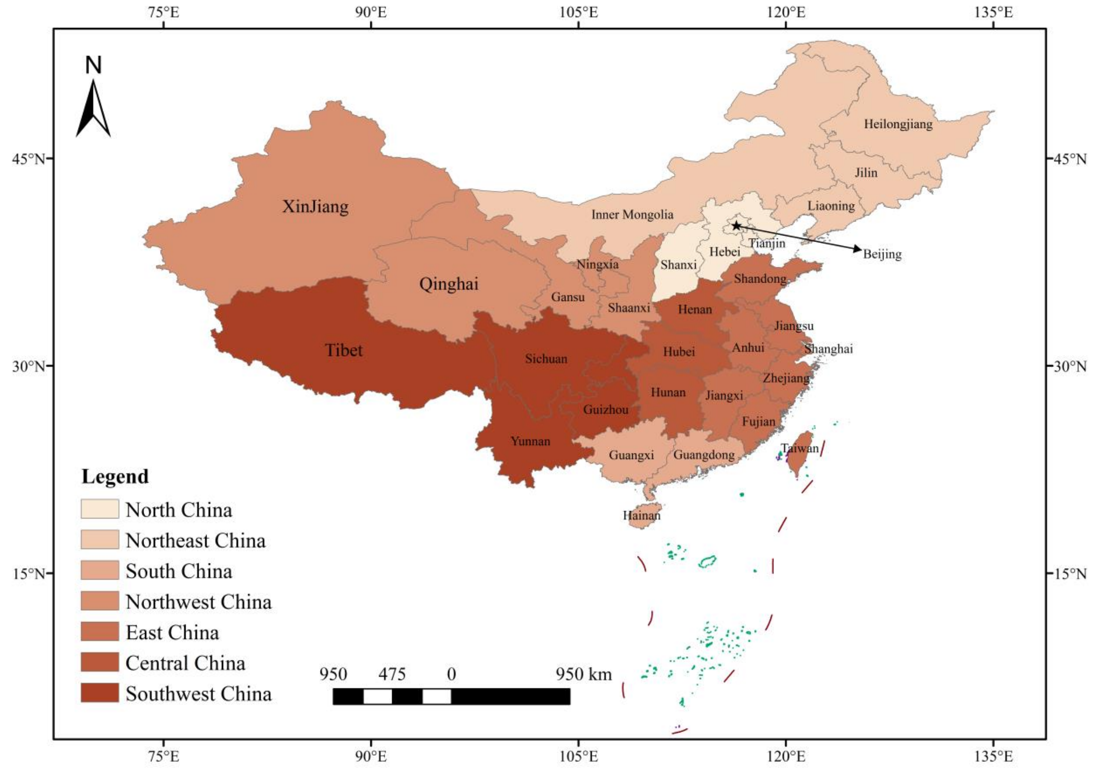

Figure 3S Distribution Map of Pepper Planting Areas in China.
